# Experimental research into the potential therapeutic effect of GYY4137 on Ovariectomy-induced osteoporosis

**DOI:** 10.1186/s11658-018-0114-0

**Published:** 2018-10-01

**Authors:** Zhong-Shi Xu, Feng Dai, Ji Chen, Meng Lv, Ji-Wu Cheng, Xiao-Ming Zhang, Bo-Wen Lin

**Affiliations:** 1 Department of Orthopedics, Second Clinical Medical College of Jinan University (Shenzhen People’s Hospital), Dongmen North Road 1017, Luohu District, Shenzhen, 518020 China; 2 Department of Radiology, Second Clinical Medical College of Jinan University (Shenzhen People’s Hospital), Shenzhen, 518020 China

**Keywords:** H_2_S, Osteoporosis, Ovariectomized, Bone metabolism, Bone mineral density

## Abstract

**Background:**

Evidence has shown that endogenous H_2_S plays an important role in the physiological and pathophysiological processes of many organs. The study aimed to explore whether exogenous H_2_S has a potential therapeutic effect on a rat ovariectomy-induced model of osteoporosis.

**Methods:**

The OVX osteoporosis model was established in female Sprague-Dawley rats by full bilateral ovariectomy. The rats were randomly divided into four groups, with the two experimental groups receiving an intraperitoneal injection of GYY4137 or sodium alendronate. The level of H_2_S in the plasma was determined and common laboratory indicators to diagnose osteoporosis, such as alkaline phosphatase (ALP) activity and the levels of osteocalcin (OCN), calcitonin, parathyroid hormone and leptin were measured. The bone mineral density (BMD) of the 4th and 5th lumbar vertebrae was measured using dual-energy X-ray absorptiometry. The maximum stress of femoral fracture was obtained through a three-point bending test of the femur.

**Results:**

The OVX osteoporosis model was successfully established. GYY4137 was injected to increase the level of H_2_S in the plasma in one group, designated OVX-GYY during the observation period (*p* < 0.05). At 12 weeks, the BMD value of the fourth lumbar vertebra in the OVX-GYY group had increased (*p* < 0.05). The BMD femur value in the OVX-vehicle group had decreased (*p* < 0.05). Bilateral ovariectomy leads to biochemical disorders related to bone metabolism and hormone levels in rat plasma (all *p* < 0.05). Ovariectomy also reduced blood calcium, blood phosphate and calcitonin, and increased parathyroid hormone and leptin. The opposite results were obtained for the groups with alendronate sodium or GYY4137 treatment (all *p* < 0.05).

**Conclusions:**

Through the slow release of H_2_S, GYY4137 did an excellent job of simulating endogenous neuroendocrine gaseous signaling molecules. Exogenous H_2_S had a regulatory effect on osteoporosis in ovariectomized rats, showing potential value for the treatment of human postmenopausal osteoporosis.

## Background

With a growing aging population in many countries, osteoporosis has become a more common clinical diagnosis. It increases bone fragility and the likelihood of fracture, which can seriously affect the physical health and quality of life of those affected, even resulting in disability and a shorter lifespan [1].

Osteoporosis is frequently found in postmenopausal women, with the diagnosis usually noted as postmenopausal osteoporosis (PMOP) [2]. Estrogen influences bone metabolism in several ways. Together with various other endogenous hormones, it maintains the balance of calcium phosphate in the plasma. An imbalance in calcium phosphate in the plasma is proven to have a relationship to osteoporosis.

One of the most common treatments for osteoporosis is exogenous hormone replacement therapy. However, this can increase the risk of tumors of the reproductive system [3]. Bisphosphonate is another first-line drug used to treat osteoporosis. It can slow down bone resorption by inhibiting the activity of osteoclasts. However, it also inhibits osteogenesis, sometimes with serious adverse effects. Long-term use can lead to jawbone osteonecrosis and long bone atypical fracture [4]. Regrouping parathyroid hormone (PTH) drugs have been shown to directly promote bone formation, but they concurrently stimulate the growth of osteoclasts, which increases bone resorption [5, 6]. Thus, finding new therapies has important clinical significance.

H_2_S is generally considered a virulent gas. In high concentrations, it inhibits cytochrome c oxidase in the mitochondria, with an even stronger effect than cyanide. However, in 1996, Abe and Kimura proposed its function as an endogenous neurotransmitter [7]. Since then, increasing evidence has shown that endogenous H_2_S plays an important role in the physiological and pathophysiological processes of many organs. It can regulate many important cell functions through signal transmission in and out of cells. It is the third most common endogenous gaseous signaling molecule after nitric oxide and carbon monoxide [8]. The level of oxidative stress in the body increases with age [9]. As a reductant, H_2_S in physiological concentrations is anti-oxidant, anti-inflammatory and anti-apoptotic [10, 11]. The concentration of endogenous H_2_S in the plasma is negatively correlated with aging speed and the level of oxidative stress [12].

It is currently commonly believed that oxidative stress plays an important role in PMOP. Estrogen could prevent osteoporosis through its anti-oxidant activity and maintain the balance of bone metabolism [13, 14]. In ovariectomy (OVX) osteoporosis models, fat peroxidation and hydrogen peroxide levels have been found to increase in rat leg muscle homogenate. When the levels of anti-oxidant enzymes decrease, excessive active oxygen produces oxidative stress, causing osteoblast damage or death, stimulating osteoclast functions and extending their lifespan, thus leading to osteoporosis [15, 16]. Similar results have been observed in humans [17, 18]. Some studies demonstrate that exogenous H_2_S can stimulate osteoblast formation and differentiation through an ERK1/2-dependent antioxidant mechanism, successfully inhibiting hydrogen peroxide-induced oxidative damage to osteoblast strain MC3T-E1 [19].

GYY413 is a new controlled-release donator of H_2_S that was developed in recent years. It slowly and continuously releases H_2_S inside the body with no toxicity or side effects. It can imitate endogenous H_2_S formation and has an edge over sodium hydrosulfide as an exogenous donor [20–22]. To explore the therapeutic effects of GYY4137 in osteoporosis, we established an OVX-induced osteoporosis rat model and used different treatments to observe the changes in the levels of H_2_S, the hormones related to bone metabolism and the serum hormones. We also determined the bone density and used a biomechanics test to explore the effects of GYY4137 on the bones.

## Methods

### Experimental animals

The animal experiments were approved Ethics Committee of Shenzhen People’s Hospital (Approval No. SZPH-160528; Approval date: 10 Mar 2016). All animal experiments were conducted according to relevant national and international guidelines. The Laboratory Animal Center of Guangdong Province provided 80 6-month old female Sprague-Dawley rats (265 ± 15 g). They were kept in a room with a temperature of 23 ± 2 °C, relative humidity of 55 ± 10%, and a regular 12-h light cycle. They were fed by designated persons and could eat and drink freely. The rats were allowed to acclimate for 1 week before grouping and osteoporosis modeling.

### Operative techniques

The 80 rats were randomly divided into four groups of 20 rats each: the sham-vehicle group; ovariectomized group with vehicle treatment (OVX-vehicle); ovariectomized group alendronate sodium treatment (OVX-ALEN); and ovariectomized group with GYY4137 treatment (OVX-GYY). OVX-ALEN and OVX-GYY were the treatment groups.

GYY4137 was purchased from Santa Cruz Biotechonology (SC-224013) and was diluted with 0.9% physiological saline. The trade name of the alendronate sodium is A115345. It was also diluted with 0.9% physiological saline.

Rats were fasted overnight with free access to water before the operation. All the rats were anesthetized with an intraperitoneal injection of chloral hydrate (Sangon Biotech) at a dose of 320 mg/kg. They were fixed on their backs, their abdomens were disinfected, and an incision was made in the middle of the abdomen. The uterus bicornis and bilateral ovaries were found, and the surrounding adipose tissue was isolated. No massive hemorrhage was allowed during the operation. After waking up, the rats were treated with penicillin (40,000 U/per rat) for 3 days to avoid infection.

For the sham-vehicle group, after the ovaries were isolated, the incision was sewn up. Along with the penicillin, the rats were injected with 1 mg/kg normal saline into the abdominal cavity. For the OVX-vehicle group, the ovariectomy was completely performed and the incision was sewn up. The OVX-vehicle group also received 1 mg/kg saline injected into the abdominal cavity. The two treatment groups also underwent the same OVX operation. The OVX-ALEN group was subcutaneously given alendronate sodium (A115345) at a dose of 1 mg/kg and the OVX-GYY group was given GYY4137 at a dose of 100 μg/kg. The frequencies of administration for all rats were once every 2 days.

At the end of the second, fourth, eighth and twelfth weeks, 5 rats were randomly selected, anesthetized via ether inhalation and euthanized with an injection of pelltobarbitalum sodium. The abdomens were incised to collect blood from the abdominal aorta. Plasma was separated from the blood and both femurs were completely removed, wrapped in gauze saturated with normal saline, marked separately, and saved in the refrigerator at − 80 °C. At the end of the eighth and twelfth weeks, lumbar vertebrae, soft tissues, and adnexa were removed. They were preserved in the same way as the femurs.

There was no accidental death during the study. All experimental steps conformed strictly to the *Guidelines for the Management and Use of Laboratory Animals* enacted by the Ministry of Science and Technology of the People’s Republic of China. The experiment duration was 12 weeks.

### Determination of the levels of serum phosphorus and calcium

Blood from the abdominal aorta was sampled and EDTANa_2_ (1 mg/ml) was added as an anticoagulant. Blood phosphate levels were determined via phosphomolybdic acid UV colorimetry using a phosphorus determination kit (Solarbio, China). Phosphate absorbance and concentration were measured with a spectrophotometer (ThermoFisher, USA). The blood calcium levels were determined via the colorimetric method using a Roche automatic biochemical analyzer (cobas 8000 c702).

### Determination of serum hormone levels

Blood from the abdominal aorta was sampled and EDTANa_2_ (1 mg/ml) was added as an anticoagulant to separate the plasma. The level of ALP was measured via the velocity method using a Roche alkaline phosphatase kit. The levels of OCN and calcitonin were determined via chemiluminescence using a Roche osteocalcin or calcitonin kit, respectively. The level of parathyrin was measured via electrochemiluminescence using a Roche parathyroid hormone detection kit and a Roche automatic biochemical analyzer. The level of leptin was determined via ELISA (enzyme linked immunosorbent assay) using a Huamei Rat Leptin LEP ELISA kit.

### Determination of the H2S level in the plasma

The improved methylene blue method was used to determine the level of H_2_S in the plasma. First, an overdose of zinc acetate solution was added to integrate free H_2_S, HS-, and S_2_- into the ZnS sediment. Then, 5 mol/l of NaOH was added to dissolve the denatured proteins. The solution was centrifuged to remove most of the proteins. An N-Dimethyl p-phenylenediamine hydrochloride solution with 0.2% N was added to completely dissolve ZnS, whereupon trichloroacetic acid was used to deposit the remaining proteins. Finally, a spectrophotometer was used to measure the level of H_2_S.

### Determination of bone density

A Hologic Double Energy X Ray 4500 W Bone Densitometer System (DEXA) was used to measure the fourth and fifth lumbar vertebrae and the BMD of both femurs from each rat.

### Determination of biomechanics

Femur samples were excised to conduct biomechanical measurements. The maximal stress during femur fracture was measured via three-point bending experiment using an ElectroForce 3200 Series II Test Instrument (BOSE).

### Hematoxylin–eosin staining

After fixation in 4% paraformaldehyde, each femur sample was decalcified, dehydrated in ethanol at incremental concentrations, washed in xylene, and implanted into histological paraffin. Finally, 5 μm slices were used to perform H&E staining.

### Statistical analysis

The experimental data are expressed as means ± standard deviation. The statistical analysis was performed with SPSS 22.0 software. Based on the homogeneity test of variance results, Student’s t test was used to compare two different groups. *p* < 0.05 was considered statistically significant.

## Results

### GYY4137 reversed the osteoporosis caused by ovariectomy

**Fig. 1:**
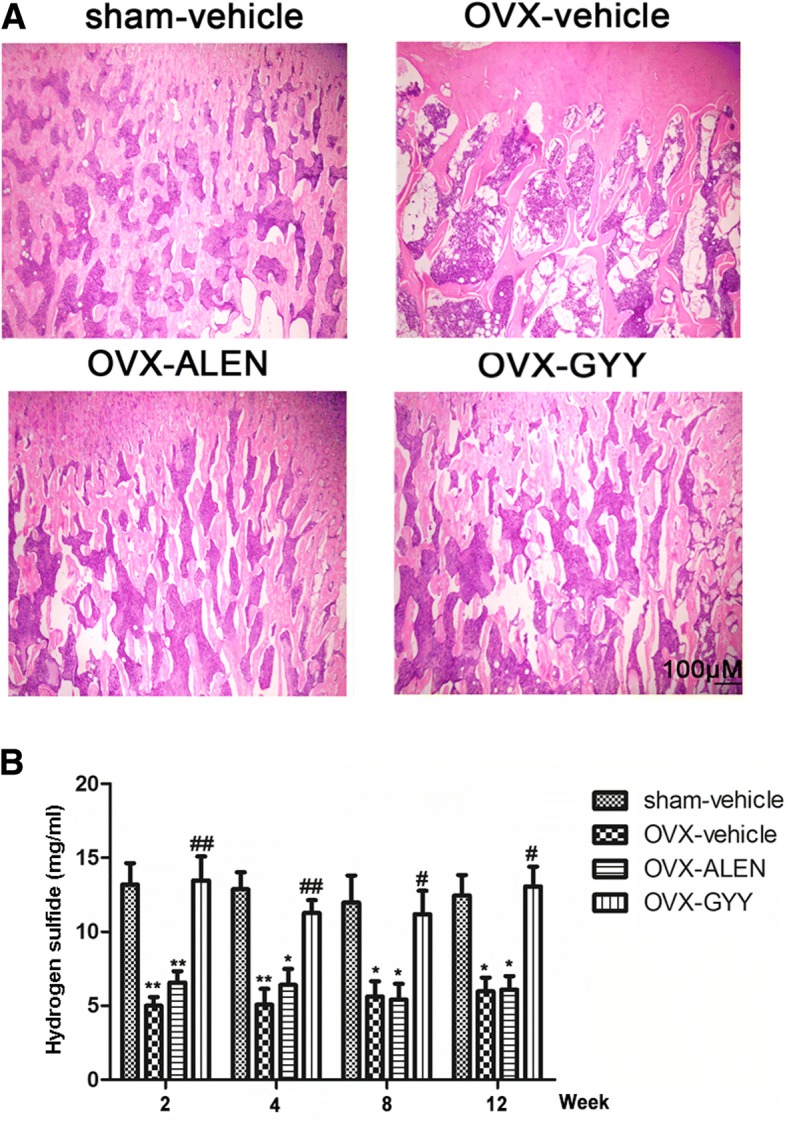
Establishment of the osteoporosis model **a** Photos of representative 5-μm slices of femurs with H&E staining (mag. 40×). **b** The results of the test for H_2_S level in the rat plasma samples. *p < 0.05 vs. sham-vehicle group; **p < 0.01 vs. sham-vehicle group; ^#^*p* < 0.05 vs. OVX-vehicle group; ^##^*p* < 0.01 vs. OVX-vehicle. OVX-vehicle: ovarectomized rats treated with vehicle; OVX-ALEN: ovarectomized rats treated with alendronate sodium; OVX-GYY: ovarectomized rats treated with GYY4137

The 5 μm H&E-stained femur slices were examined under a microscope (mag. 40×). The OVX-vehicle group showed obvious osteoporosis and the trabecular bone was broken. The trabecular bones of the sham-vehicle, OVX-ALLEN and OVX-GYY groups were normal with no obvious osteoporosis (Fig. 1a). Compared with the sham-vehicle group, the levels of H_2_S in the OVX-vehicle and OVX-ALLEN groups had decreased significantly, but the level in the OVX-GYY group was not significantly different (Fig. 1b).

### GYY4137 regulates the level of hormones related to bone metabolism in the OVX model

**Fig. 2:**
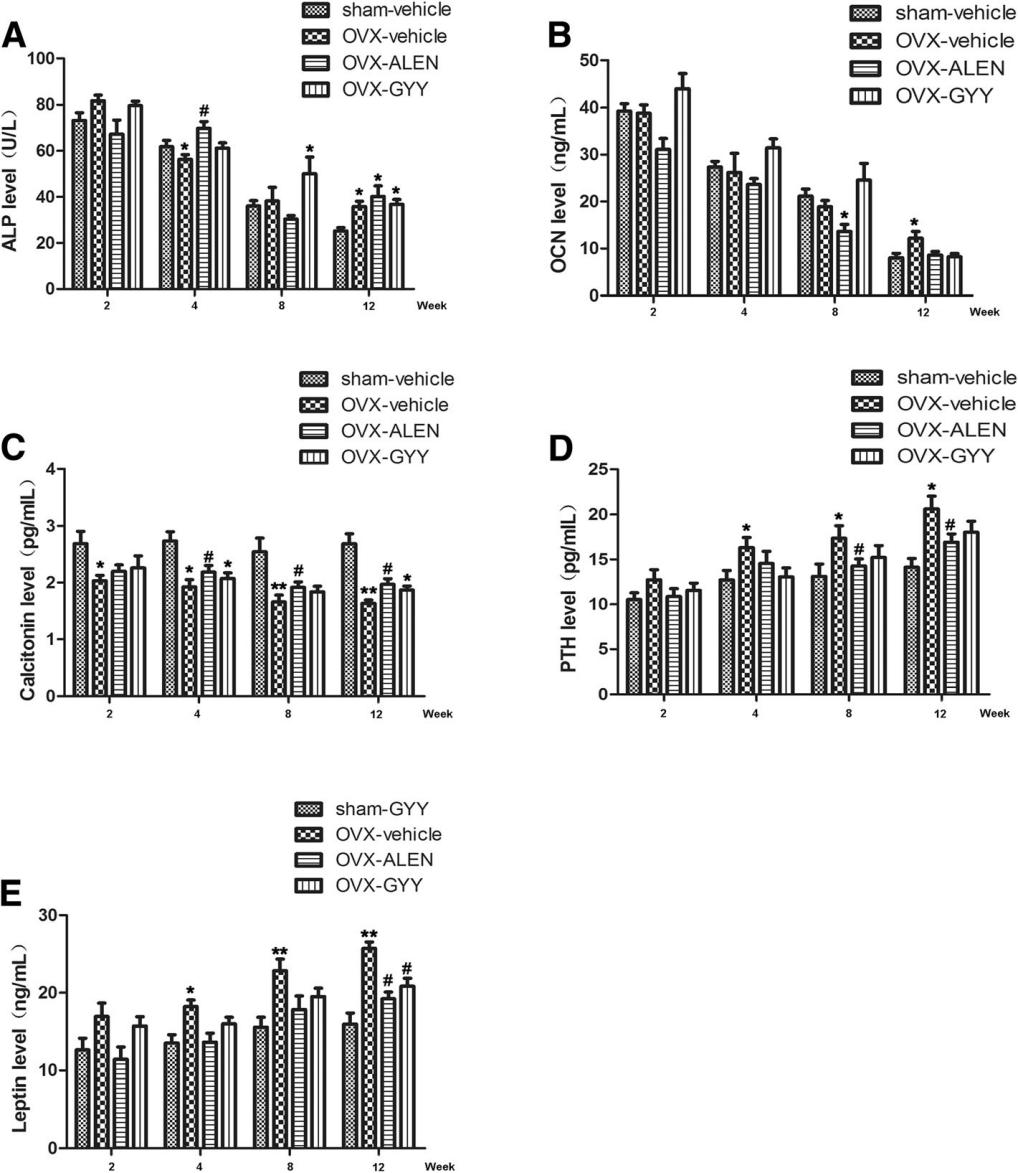
The effect of GYY4137 on the hormone levels in the rat plasma related to bone metabolism in the OVX model. The levels of **a** alkaline phosphatase, **b** osteocalcin, **c** calcitonin, **d** parathyrin and **e** leptin were assessed in all four groups of rats. *p < 0.05 vs. sham-vehicle group; **p < 0.01 vs. sham-vehicle group; ^#^*p* < 0.05 vs. OVX-vehicle group; ^##^*p* < 0.01 vs. OVX-vehicle. OVX-vehicle: ovarectomized rats treated with vehicle; OVX-ALEN: ovarectomized rats treated with alendronate sodium; OVX-GYY: ovarectomized rats treated with GYY4137

The most common indicators associated with a clinical diagnosis of osteoporosis and follow-up observations were chosen, including ALP, OCN, calcitonin, parathyrin, and leptin. Figure 2a and b show the results for the ALP and OCN levels. In detail, compared with the sham-vehicle group, the ALP level in the OVX-vehicle group significantly had decreased by week 4 (56.4 ± 4.33 vs. 61.8 ± 6.14, *p* < 0.05). After 8 weeks, the ALP level in the OVX-GYY group had significantly increased, but failed to recover to the 2-week level (50 ± 16.37 vs. 36 ± 5.33, *p* < 0.05). After 12 weeks, the ALP level in the OVX-vehicle, OVX-ALEN and OVX-GYY groups had significantly increased (35.8 ± 5.26, 40.2 ± 10.32, 36.8 ± 4.65 vs. 25.2 ± 3.19, all *p* < 0.05). However, ALEN treatment significantly had decreased the OCN level by week 8 (13.66 ± 3.34 vs. 21.15 ± 3.40, *p* < 0.05), while the ONC level after 12 weeks was significantly higher in groups that had undergone the OVX operation (12.18 ± 3.24 vs. 8.03 ± 2.1, *p* < 0.05).

PTH and leptin levels significantly increased, as shown in Fig. 2d and e. After 4, 8 and 12 weeks, the PTH level had increased in groups that had undergone the OVX operation (16.32 ± 2.53 vs. 12.72 ± 2.35; 17.36 ± 3.14 vs. 13.12 ± 3.06; 20.64 ± 3.16 vs. 14.14 ± 2.16, all *p* < 0.05) compared to the sham group. Similarly, the leptin level rose in the OVX group at 4, 8 and 12 weeks (18.2 ± 1.93 vs. 13.5 ± 2.44; 22.84 ± 3.37 vs. 15.56 ± 2.85; 25.72 ± 1.80 vs. 15.96 ± 3.18, all *p* < 0.05).

The calcitonin level only showed a relationship with time for the OVX-vehicle group. No other group showed such a relationship during the 12 weeks (Fig. 2c).

After the ovariectomy, the calcitonin level decreased over time (Fig. 2c). With alendronate sodium and GYY4137 treatment, the levels of calcitonin, parathyrin, and leptin were regulated and balanced (Fig. 2c, d, e). The effects of alendronate sodium on calcitonin and parathyrin were significant, respectively from week 4 and 8 (*p* < 0.05; Fig. 2c and d). The effect of alendronate sodium and GYY4137 on leptin levels became significant from week 12 (*p* < 0.05; Fig. 2e).

### The effect of GYY4137 on the amount of calcium and phosphorus in ovariectomized rat plasma

**Fig. 3:**
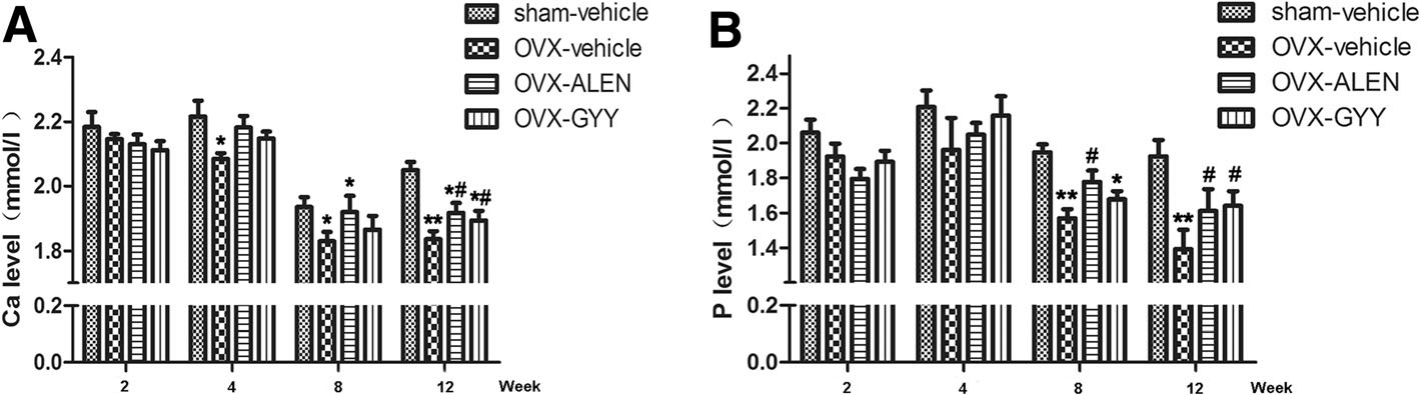
The effect of GYY4137 on the amount of calcium and phosphorus in the plasma of ovariectomized rats. **a** The amount of calcium in the plasma. **b** The amount of phosphorus in the plasma. *p < 0.05 vs. sham-vehicle group; **p < 0.01 vs. sham-vehicle group; ^#^*p* < 0.05 vs. OVX-vehicle group; ^##^*p* < 0.01 vs. OVX-vehicle. OVX-vehicle: ovarectomized rats treated with vehicle; OVX-ALEN: ovarectomized rats treated with alendronate sodium; OVX-GYY: ovarectomized rats treated with GYY4137

Compared with the sham-vehicle group, the OVX-vehicle group showed a significantly reduced level of calcium in the blood after 4 weeks (2.09 ± 0.04 vs. 2.23 ± 0.11, *p* < 0.05), which became more evident after 12 weeks (1.83 ± 0.06 vs. 2.05 ± 0.05, *p* < 0.05; Fig. 3a). The OVX-vehicle group began to show a reduced level of phosphorus after 8 weeks (1.57 ± 0.12 vs. 1.95 ± 0.10, *p* < 0.05; Fig. 3b). After intervention with alendronate sodium and GYY4137, the phosphorus in the blood recovered to some degree (Fig. 3a and b).

### GYY4137 relieved ovariectomy-induced osteoporosis and the decline in bone mineral density

Compared with the sham-vehicle group, the BMD of the fourth and fifth lumbar vertebrae in the OVX-vehicle group had decreased by weeks 8 and further by week 12 (0.195 ± 0.01 vs. 0.244 ± 0.02, 0.193 ± 0.02 vs. 0.239 ± 0.01, 0.182 ± 0.02 vs. 0.222 ± 0.02, 0.180 ± 0.02 vs. 0.232 ± 0.03, all *p* < 0.05). The differences were statistically significant.

**Fig. 4:**
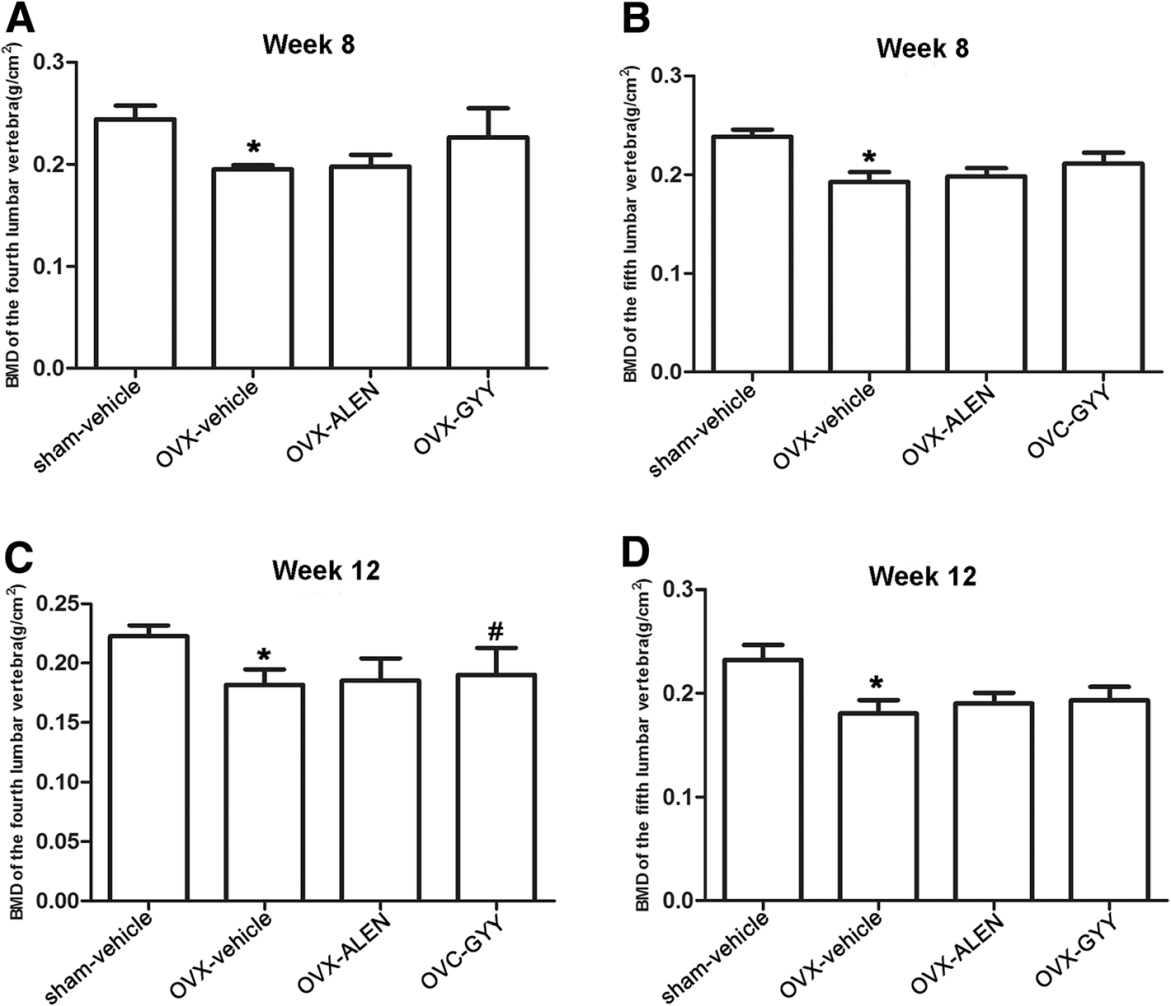
The results of the bone density test for the lumbar vertebrae. The BMD measurements are for: the fourth lumbar vertebra in week 8 (**a**); the fifth lumbar vertebra in week 8 (**b**); the fourth lumbar vertebra in week 12 (**c**); the fifth lumbar vertebra in the week 12 (**d**). The results for the OVX-vehicle group were compared with those for the sham-vehicle group (*p* < 0.05) and OVX-GYY group (*p* < 0.05). *p < 0.05 vs. sham-vehicle group; ^#^*p* < 0.05 vs. OVX-vehicle group. OVX-vehicle: ovarectomized rats treated with vehicle; OVX-ALEN: ovarectomized rats treated with alendronate sodium; OVX-GYY: ovarectomized rats treated with GYY4137

Treatment with GYY4137 relieved the decline in BMD over the 12 weeks (0.19 ± 0.04 vs. 0.182 ± 0.02, *p* < 0.05), with a better therapeutic effect than found in the OVX-ALEN group (Fig. 4).

### GYY4137 improves the maximum bone stress of ovariectomy-induced osteoporosis

**Fig. 5:**
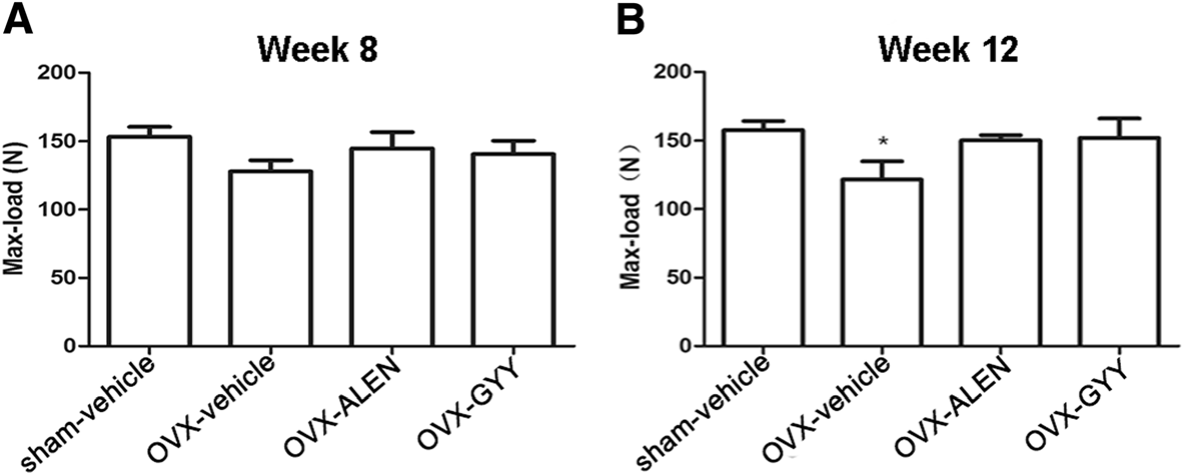
The results of the biomechanical maximum load experiment. **a** The OVX model in week 8. **b** The OVX model in the week 12. The results for the OVX-vehicle group were compared with those for the sham-vehicle group (*p* < 0.05) and OVX-GYY group (*p* < 0.05). *p < 0.05 vs. sham-vehicle group. OVX-vehicle: ovarectomized rats treated with vehicle; OVX-ALEN: ovarectomized rats treated with alendronate sodium; OVX-GYY: ovarectomized rats treated with GYY4137

The maximum stress of fracture of the femur was tested with the three-point bending test (Fig. 5). Relevant outliers were removed based on Grubbs’ criterion. The results showed that after 12 weeks, the maximum bone stress of the OVX-vehicle group had decreased. OVX osteoporosis modeling changes the maximum biomechanical load of the thighbone in a manner consistent with the effects of osteoporosis. Rats treated with alendronate sodium or GYY4137 showed no significant differences in the maximum biomechanical load (*p* > 0.05) compared with the sham-vehicle group. The OVX-ALEN and OVX-GYY groups also failed to show a significant difference (p > 0.05; Fig. 5a and b). The differences were statistically significant.

## Discussion

As an endogenous gaseous signaling molecule, H_2_S is a hot research topic. Multiple investigations have verified its important role in the physiological and pathological processes of many tissue systems, including the visceral, cardiovascular, skeletal and nervous systems. For example, it can protect cardiomyocytes from ischemic disease [23]. Reduction of endogenous H_2_S can accelerate atherosis [24]. It can also weaken the protection against oxidative stress in the nervous system [25].

Estrogen promotes bone formation, which is mainly mediated by ER receptors on osteoblasts [26, 27]. It also inhibits bone absorption through the apoptosis of osteoclasts, induced by its ER receptors [28, 29]. Normally, bone metabolism depends on homeostasis and the balance of osteoblast-mediated bone formation and osteoclast-mediated bone absorption [30]. The levels of calcium and phosphorus in the plasma are maintained in a dynamic balance with their levels in the bone, with the plasma levels reflecting the metabolism of bone tissue and the absorption–reabsorption rate of calcium and phosphorus by the small intestine and kidneys.

In this study, we investigated the effects of estrogen deficiency on the levels of H_2_S, calcium and phosphate in rat plasma, on the macroscopic mechanics and physics of the bones, and on related hormone secretion. We also studied how GYY4137 and alendronate sodium affected these parameters. The four groups of rats were the sham-vehicle group; ovariectomized group with vehicle treatment (OVX-vehicle); ovariectomized group alendronate sodium treatment (OVX-ALEN); and ovariectomized group with GYY4137 treatment (OVX-GYY).

The OVX-GYY group showed no significant difference compared to the sham-vehicle group in terms of macroscopic mechanics and physics. Exogenous GYY was proven to raise the level of H_2_S in rat plasma and improve the imbalances in calcium and phosphate caused by the lack of ovaries. It also influenced the leptin level and raised the density of the lumbar vertebrae and the maximum stress of the thighbone. The influence of estrogen deficiency on bone metabolism was partially neutralized. H&E staining and microscopic observation also proved that with the intervention of GYY, the structure and amount of trabecular bone in the OVX-GYY group was not significantly different than the results for either the sham-vehicle or OVX-ALEN groups.

Other than directly acting on bone metabolism, estrogen can increase the secretion of calcitonin, inhibit the secretion of parathyrin, promote the activation of vitamin D in the kidney, and increase calcium absorption in the small intestine through a calcium-regulating hormone.

Calcitonin is a kind of polypeptide hormone secreted by thyroid C cells. It acts on calcitonin receptors to inhibit the activation of osteoclasts, stimulate the proliferation of osteoblasts, and prevent apoptosis [31, 32]. Calcitonin can also indirectly inhibit the activation of parathyroid and vitamin D and reduce the level of calcium in the blood. Its levels have no association with age, so the basic values of calcitonin and metabolic rate show no significant change for people with osteoporosis, which concurs with the result in our study.

Parathyrin is secreted by the parathyroid in a pulsatile way, acting on the parathyrin receptors of osteoblasts to enhance bone formation. The abnormal secretion of parathyrin, such as the constant abundant secretion caused by hyperparathyroidism, can increase the activity of osteoclasts and result in bone resorption and osteoporosis. Our study demonstrated that the parathyrin level decreases over time, which might have something to do with the reduction in estrogen in the plasma.

In our OVX-ALEN group, alendronate sodium regulated calcitonin and parathyrin. However, GYY had no significant regulatory effect on these two hormones in the OVX-GYY group.

Leptin, a body-wide hormone secreted by the white adipose tissue, has a more complex regulatory relationship with bone metabolism. It is generally believed that it acts on hypothalamic long receptors (OB-Rb) to regulate bone metabolism through the neuroendocrine axis. It can also directly regulate bone metabolism through short receptors around the bone tissue. Thomas, et al., [33] tested BMD and the serum leptin level of pre- and post-menopausal women and men. They found that the level of serum leptin in women was negatively associated with BMD, while there was no significant association in men. In this study, we observed that H_2_S might help regulate the level of leptin.

Approximately 50% of ALP in the plasma comes from osteoblasts. OCN is an active polypeptide directly secreted and expressed by osteoblasts. They both reflect the activity and state of osteoblasts and represent bone formation and reconstruction [34]. Interestingly, neither alendronate sodium nor GYY4137 exerted significant regulatory effects on ALP or OCN in the OVX groups. This demands further study.

The regulation of bone metabolism has many interactive signaling pathways at the molecular level, and these play important roles in bone formation and absorption [35]. The bone morphogenetic protein (BMP) Smads pathway and the Wnt/β-catenin pathway mainly affect bone formation, while the pathway involving osteoprotegerin (OPG), nuclear factor κB receptor activator of the NF-kB ligand (RANKL) and nuclear factor κB receptor activator in NF-Kb (RANK) mainly affects bone absorption. Some research has shown that H_2_S produced by mesenchymal stem cells can regulate bone differentiation, and that the reduction of H_2_S can reduce the thiolation of calcium channels, influencing the internal flow of the Ca^2+^ pathway in the cytoderm. This reduces the differentiation of mesenchymal stem cells to osteoblasts – a process that is regulated by the Wnt/β-catenin signal pathway and mediated by PKC and ERK [36]. H_2_S can also interact with Osterix by affecting the important transcription factor nuclear-binding protein (Cbfα1/Runx2) in the BMP/Smads pathway to promote osteocyte proliferation and differentiation. In addition, it can further activate the expression of specific osteoblast factors (ALP, type I collagen, and OCN) to promote bone formation [16]. The BMP/Smads and Wnt/β-catenin signal pathways can mutually regulate each other [37].

In some experiments, H_2_S has also been observed to mediate oxidative stress injury, promote the proliferation and differentiation of osteoblasts, and alleviate inflammation through a mechanism dependent on EPK1/2 and p38 in the MAPK pathway [38]. Osteoblasts regulate the proliferation and differentiation of osteoclasts, mainly through the OPG/RANKL/RANK signaling pathway, while H_2_S might regulate the activity of osteoclasts by increasing OPG expression and reducing RANKL expression in bone tissue [39–41]. Gambari, et al., [42] also observed that H_2_S could directly inhibit the differentiation of osteoclasts through a mechanism dependent on NRF2. These statements are in accord with the results of this study, indicating the benefit of H_2_S to bone metabolism and its potential to improve the sclerotin structure.

Exogenous H_2_S can intervene and regulate osteoporosis in ovariectomized rats. In some cases, it could even cure osteoporosis caused by ovariectomy [43]. The effect of H_2_S on osteoporosis is significantly dependency on dosage. Further studies on the effect of the H_2_S concentration on bone metabolism are needed. The concentration of sulfide ions in the blood of people with H_2_S poisoning has been reported to be 3–995 μM [44]. The physiological concentration is quite close to the minimal concentration for H_2_S poisoning. At high concentrations, it can even induce lipopolysaccharide-mediated inflammation for rats and synchronously increase the H_2_S concentration in the plasma of patients with septic shock [45]. The effect of exogenous H_2_S on important tissues and organs needs to be studied in terms of method, speed, and concentration of drug administration.

In summary, ovariectomized rats were used as subjects in this study to explore the association between the level of endogenous H_2_S in the plasma and osteoporosis. Through intraperitoneal injection of GYY413 as the supplement of endogenous H_2_S, we showed that raising and maintaining the physiological level of H_2_S in the plasma could regulate the metabolic balance of calcium and phosphorus. We also looked at the impact on common clinical indexes of bone metabolism: the hormones OCN, calcitonin, alkaline phosphatase, parathyrin and leptin. Our results suggest that GYY4137 is a better intervention than alendronate sodium and that it has a certain therapeutic effect on ovariectomy-induced osteoporosis.

### Limitations

The therapeutic effects of GYY4137 in a rat model of osteoporosis is one of our study limitations. We did not explore the therapeutic effects of GYY4137 in the clinic, so the result may differ in humans. In addition, the molecular mechanism of GYY4137 action needs to be explored. Any potential bias would also be considered in further study.

## Conclusion

Exogenous H_2_S can inhibit osteoporosis caused by lack of estrogen, increase the maximum stress of bone, and reduce the occurrence of osteoporosis-related fracture complications. This study may indicate a direction for future study on a therapeutic target for postmenopausal osteoporosis.

## Abbreviations

ALP: Alkaline phosphatase
BMD: Bone mineral density
BMP: Bone morphogenetic protein
OCN: Osteocalcin
PMOP: Postmenopausal osteoporosis
PTH: Parathyroid hormone
